# Tailoring micro/nano-fibers for biomedical applications

**DOI:** 10.1016/j.bioactmat.2022.04.016

**Published:** 2022-04-25

**Authors:** Bin Kong, Rui Liu, Jiahui Guo, Ling Lu, Qing Zhou, Yuanjin Zhao

**Affiliations:** a Department of Cardio-Thoracic Surgery, Institute of Translational Medicine, Nanjing Drum Tower Hospital, The Affiliated Hospital of Nanjing University Medical School, 210008, Nanjing, China; b Department of Otolaryngology Head and Neck Surgery, Jiangsu Provincial Key Medical Discipline, Nanjing Drum Tower Hospital, The Affiliated Hospital of Nanjing University Medical School, 210008, Nanjing, China; c State Key Laboratory of Bioelectronics, School of Biological Science and Medical Engineering, Southeast University, 210096, Nanjing, China; d Institute for Stem Cell and Regeneration, Chinese Academy of Science, 100101, Beijing, China

**Keywords:** Nano/micro fibers, Bioprinting, Electrospinning, Microfluidic spinning, Near field electrospinning, Biomedical field

## Abstract

Nano/micro fibers have evoked much attention of scientists and have been researched as cutting edge and hotspot in the area of fiber science in recent years due to the rapid development of various advanced manufacturing technologies, and the appearance of fascinating and special functions and properties, such as the enhanced mechanical strength, high surface area to volume ratio and special functionalities shown in the surface, triggered by the nano or micro-scale dimensions. In addition, these outstanding and special characteristics of the nano/micro fibers impart fiber-based materials with wide applications, such as environmental engineering, electronic and biomedical fields. This review mainly focuses on the recent development in the various nano/micro fibers fabrication strategies and corresponding applications in the biomedical fields, including tissue engineering scaffolds, drug delivery, wound healing, and biosensors. Moreover, the challenges for the fabrications and applications and future perspectives are presented.

## Highlights

• The widely used nano/micro fibers fabrication strategies are comprehensively reviewed.
• Focus on the application of nano/micro fibers in the biomedical fields.
• Summarize the challenges for the nano/micro fibers fabrication strategies and applications and future perspective.

## Graphical abstract

**Figure:**
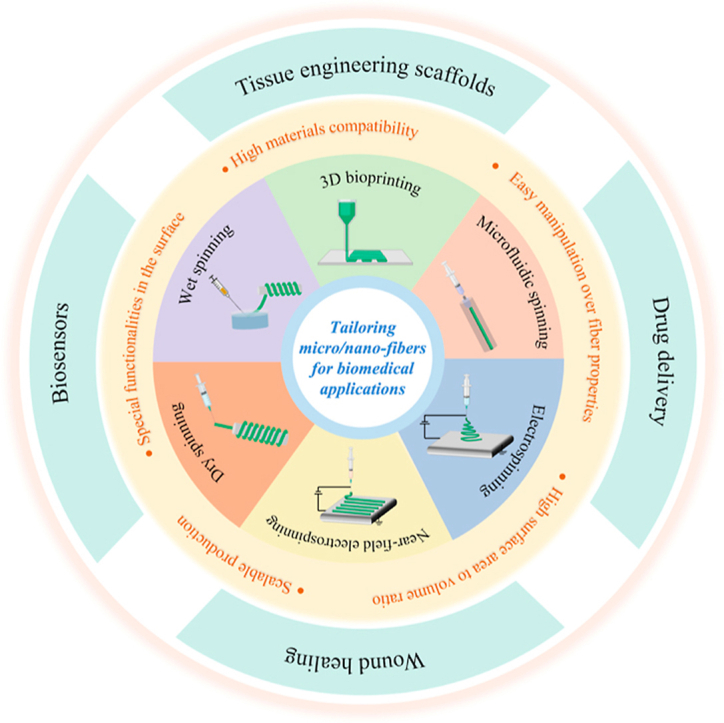

## 1 Introduction

After evolving and naturally selecting for billions of years, a large variety of novel materials have appeared in nature with attractive structures, especially the materials with the nano- or microscale that were imparted with fascinating and special functions and properties, such as structural colors [1,2], superconductivity [3,4], excellent mechanical strength [5,6] and strong adhesion [7,8]. Over the past few decades, nano/micro-fabrication technologies, which mainly focus on the operation of materials with the dimensions of nano- or microscales, such as soft lithography [9], self-assembly [10], and nano/micro fibers fabrication strategies [11,12], have been well developed and extensively used in various science and engineering research fields, such as energy [13,14], cosmetics [15], electronics [16,17], tissue engineering and regenerative medicine [[18], [19], [20], [21]]. The materials fabricated by these nano/micro-fabrication technologies have been studied and reported as the form of nano/micro fibers [19], particles [22], rods [23], spheres [24], gels [25], and sponges [26], among them, nano/micro fibers have been the research cutting edge and hotspot in the area of fiber science with the rapid development of the techniques for nano/micro fibers.

Nano/micro fibers can be easily and controllably fabricated by various strategies due to the rapid progress of material manufacturing technology, including microextrusion-based bioprinting [27], microfluidics spinning [28], wet spinning [29], dry spinning [30], electrospinning [31], near field electrospinning [32] and others [33,34]. As we know, when the diameter of the fibers decreases from the macroscopic scales to the micro or nanometers, amazing and special physiochemical properties will appear, such as the enhanced mechanical strength, high surface area to volume ratio, and special functionalities shown in the surface [35]. Due to these outstanding and special characteristics, nano/micro fibers have been widely used in diverse and important areas, including sensors, micromotors, infiltration, wettability manipulation, energy storage, catalyst, tissue engineering, drug delivery and wound healing [36]. Through the control over the fabrication devices and processing parameters, a variety of nano/micro fibers with desired fascinating morphologies could be developed, such as helical fibers, core/shell fibers, Janus fibers, porous fibers, tubular fibers, necklace-like fibers and straight fibers with beads, which could impart the fibrous scaffolds with more advanced functions. Besides, the fabrication technologies would facilitate the incorporation of bioactive functional ingredients within the fibers, such as cells, growth factors and black phosphorus, and could realize the control over the spatial and temporal distribution of these components to exhibit distinctive and special features.

**Fig. 1:**
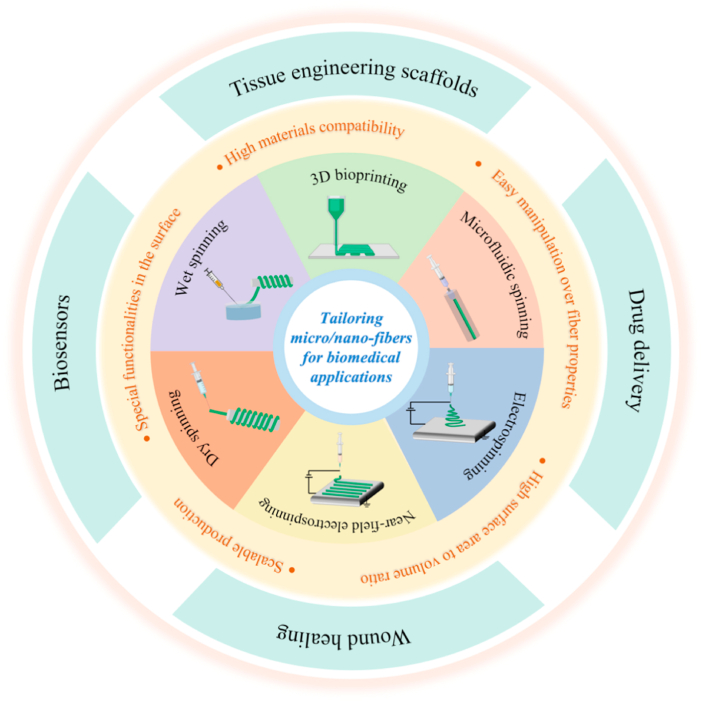
Schematic diagram of the nano/micro-fibers fabrication techniques and biomedical applications.

In this review, we present various nano/micro fibers fabrication technologies and their applications in the biomedical fields, as shown in Fig. 1. The review is divided into four sections. In Section 2, we give a detailed description of the different nano/micro fibers fabrication technologies and their recent development in the fiber materials, morphologies, and functions. In Section 3, we present the applications of nano/micro fibers and fiber-based composite materials in several biomedical fields, including tissue engineering scaffolds, drug delivery, wound healing, and biosensors. Finally, in Section 4, we provide the general conclusion of this review and discuss the present challenges and future opportunities of the nano/micro fibers fabrication technologies and nano/micro fibrous materials.

## 2 Nano/micro-fibers fabrication techniques

**Fig. 2:**
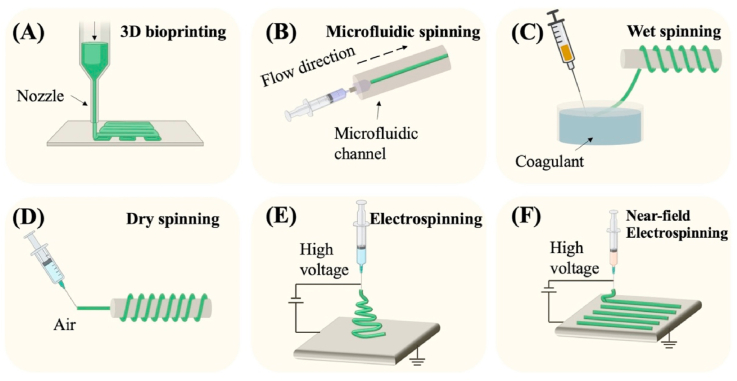
The schematic illustration of different nano/micro-fibers fabrication techniques. (A) 3D bioprinting. (B) microfluidic spinning. (C) wet spinning. (D) dry spinning. (E) electrospinning. (F) near-field electrospinning.

Various technologies have been developed to generate nano/micro fibers derived from natural or synthetic materials, including microextrusion-based three-dimensional (3D) bioprinting, microfluidic spinning, wet spinning, dry spinning, electrospinning, and near field electrospinning (Fig. 2). In this part, we will introduce these technologies respectively. The fiber diameter, fiber morphology, advantages, and disadvantages of each nano/micro fibers fabrication technique are summarized in Table 1.

**Table 1:** Summary and comparison of diverse nano/micro-fibers fabrication techniques.

Techniques	Fiber diameter	Fiber morphology	Advantages	Disadvantages
3D bioprinting	Hundreds of microns	Single fiber with solid, hollow, core−shell structures and heterogenous components	High bioink compatibility; Thick vertical structure; Capable of multiple polymerization mechanisms	Potential nozzle clog; Reduced cell viability induced by nozzle shear; Low printing resolution
Microfluidic spinning	Hundreds of nanometers to hundreds of microns	Single-fiber with solid, hollow, core−shell, Janus, multicompartmental, helical, and patterning structures	Spatiotemporal control over the fiber composition, geometry, and size; Mild process condition; Capable for the fabrication of cell-laden hydrogels	Slow fabrication process and time-consuming; Potential nozzle clog; Challenge of scale-up production
Wet spinning	Tens of microns to over one millimeter	Single fiber	Fast and efficient fabrication process; Capable for the fabrication of cell-laden hydrogels; High porosity and large pore size	Limited cell encapsulation induced by long chemical exposure time; Poor mechanical strength
Dry spinning	Several microns to hundreds of microns	Single fiber	Fast and efficient fabrication process; Mild process condition; Free from harsh solvent	Poor mechanical strength
Electrospinning	Microns to submicron/nanometers	Single fiber with porous, solid, hollow, core-shell structures; Aligned/random pattern; nonwoven mats	Diverse polymers compatible; Ability to easily fabricate nanofibers; High surface area-to-volume ratio; Easy manipulation of fiber properties; Great material handling; Scalable production	Requirement of high voltage; Solvent removal required
Near-field electrospinning	Several microns to hundreds of microns	Single fiber; layer-by-layer sequence; patterned matrix	Fabrication of micro-patterned architectures; Easy manipulation of fiber properties	Mass loss and viscosity decrease affect mechanical properties

**Table 2:** The most commonly used bioinks in microextrusion-based 3D bioprinting.

Material	Crosslinking mechanism	Printing system	Advantages	Disadvantages	Applications
Collagen	Thermal or PH mediated	Pneumatic	Excellent biological properties, good bioprinting ability	Low viscosity, slow polymerization, and poor mechanical strength	Cell attachment and 3D tissue formation
Fibrin	Enzymatic	Pneumatic	Fast gelation, promotion of new vessel formation	Poor mechanical strength and rapid degradation	Wound healing
Gelatin	Thermal	Pneumatic and mechanical	Good biocompatibility	Unstable and poor printability	Promoting cell survival and function
GelMA	Photopolymerization	Pneumatic	Good biocompatibility, improved mechanical strength	Slow gelation	Wound dressing
HAMA	Photopolymerization	Pneumatic	Good biocompatibility, improved mechanical strength	Slow gelation	Tissue engineering
Alginate	Ionic	Pneumatic	Good biocompatibility and printability, fast gelation	Poor cell attachment	Bioprinting of tissues/organs
Chitosan	Ionic or covalent	Pneumatic	Antibacterial	Poor mechanical stability and slow gelation	Drug delivery
Agarose	Thermal	Pneumatic and mechanical	High mechanical strength, nonimmunogenic	Poor cell adhesion	Tissue engineering
PEO	Ionic, covalent, or physical	Pneumatic	Good biocompatibility	Low cell adhesion and poor mechanical strength without modification	Soft tissue models
Pluronic F-127	Thermal	Pneumatic and mechanical	High printability, nonimmunogenic	Poor mechanical strength, slow gelation	Tissue engineering

### 2.1 Microextrusion based 3D bioprinting

**Fig. 3:**
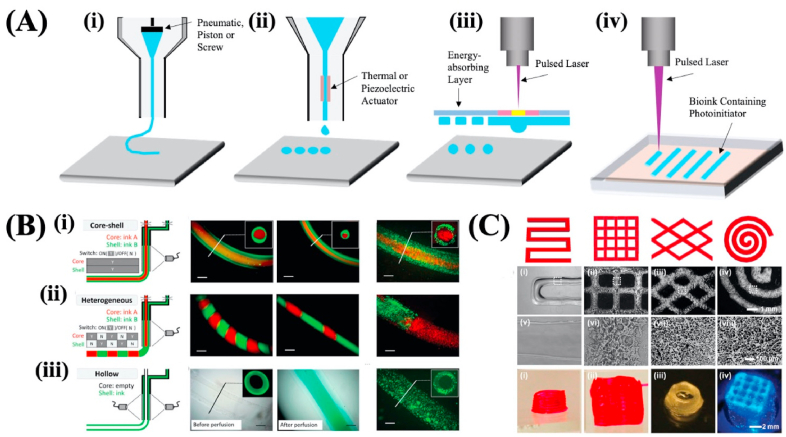
(A) The schematic illustration of (i) microextrusion-based bioprinting; (ii) inkjet-based bioprinting; (iii) laser-assisted bioprinting and (iv) stereolithography. (B) Fabrication of (i) core-shell; (ii) heterogenous; (iii) hollow microfibers via microextrusion-based bioprinting. Reproduced with permission from Ref. [38]. Copyright 2016 Wiley-VCH Verlag GmbH & Co. KGaA, Weinheim. (C) Microextrusion bioprinting scaffolds with diverse structures using two-phase emulsion bioinks. Reproduced with permission from Ref. [51]. Copyright 2018 Wiley-VCH Verlag GmbH & Co. KGaA, Weinheim.

The term 3D printing was originally put forward by Charles W. Hull in 1986 [37], which was also known as stereolithography and characterized by the layer-by-layer deposition of materials to fabricate a 3D construct with a specific structure and morphology. With the advantages of various materials compatibility and customization, 3D printing has been commonly used in the area of food, art, space, architecture, and biomedicine. And recent years, 3D bioprinting was developed when applied in the field of biomedicine, which combines the approaches of engineering with cell biology and materials science, to print bioinks with living cells and active biomolecules through the layer by layer deposition and finally form 3D scaffolds to mimic the native tissues or organs [38]. There are three most widely used 3D bioprinting approaches, including microextrusion-based bioprinting [39], inkjet-based bioprinting [40], laser-assisted bioprinting [41], and stereolithography [42] (Fig. 3A). Among these approaches, microextrusion-based bioprinting is the most commonly used technique since it's affordable, versatile, and capable to construct complicated and hollow scaffolds [43]. Besides, microextrusion-based bioprinting can easily and continuously print filaments or fibers, and in the following section, we will discuss this method in detail.

Microextrusion based bioprinting system is comprised of a fluid dispensing device to extrude bioinks through a microscale nozzle, an automated robotic system to accurately deposit materials layer by layer which is controlled by a computer, with the consistent movement of print heads along the x-, y-, and z-directions to construct any desired 3D structures using cylindrical filaments or fibers [44]. The continuous deposition of the fibers can lead to superior structural integrity. Besides, the customers can load computer-aided design (CAD) files that are derived from MRI or CT scanning images in the computer to print the specific structures automatically [45,46].

The commonly utilized driving force in the fluid dispensing device includes pneumatic, and mechanical (piston or screw) based systems (Fig. 3A). The pneumatic-based system regulates the extrusion flow by controlling compressed air using a valve free or a value-based configuration [43], and bioinks with different viscosity have different flow-air pressure relationships, therefore, the related parameters need to be calibrated when changing different bioinks. Generally, bioinks with viscosity ranging from 30 to over 6 × 10^7^ mPa s can be dispensed through bioprinting nozzles driven by pneumatic force [47,48]. The pneumatic-based system can supply driving force with a wide range, and can precisely control the flow in the start and ending position. However, the pneumatic-based system needs extra pneumatic actuators which can result in a more complicated system and large noise. The motor is used in the piston-based system to drive the movement of the piston to further dispense the bioinks, and the rotation speed of the motor directly determines the flow of the bioinks [49]. The piston-based system is simple and easy to operate, but it's not beneficial for dispensing bioinks with high viscosity which can lead to the stress accumulation between the piston and the bioinks and further influence the printing accuracy. In the screw-based system, the screw is rotated in the container and the bioinks within will be extruded out along the thread grooves of the screw. The large driving force and precise control make this approach very suitable for dispensing the bioinks with higher viscosity (>6 × 10^7^ mPa s) [47,50].

After decades of development and research, various bioinks have been explored that can be used in extrusion-based bioprinting, and according to biocompatibility, they can be classified as natural materials derived bioinks and synthetic materials derived bioinks. Table 2 shows the polymers widely used as microextrusion-based 3D bioprinting bioinks and their advantages, disadvantages, and applications.

The commonly used natural materials in extrusion-based bioprinting include collagen, gelatin, fibrin, chitosan, matrigel, hyaluronic acid (HA), alginate or alginic acid, and agarose. Collagen is the most widely used material when constructing a tissue-engineered scaffold since it appears in most human tissues or organs as the primary composition of extracellular matrix (ECM), which exhibits excellent biocompatibility and low immunogenicity. Rat tail collagen type I has been demonstrated that can form hydrogels when the PH of the collagen solution is neutralized to 7–7.4 under the temperature of 37 °C for 20–30 min. The earliest report about the extrusion-based bioprinting of collagen appears in 2004, and the researchers used a pneumatic-based bioprinting system to successfully fabricate collagen hydrogel fibers with bovine aortic endothelial cells [52]. Although collagen is printable and has excellent biological properties, the low viscosity, slow polymerization, and poor mechanical strength usually cannot support the 3D structure and limit its application in tissue engineering. Thus, many researchers have attempted to supplement additional components or polymers to obtain better printing capability and mechanical strength. Pluronic® F-127 was used to be blended with collagen type I, and the hybrid solutions with rat-derived bone marrow-derived stem cells (BMSCs) were printed using a thermally controlled extrusion-based bioprinting device. BMSCs maintained high viability, good proliferation, and can spread within the fibrous construct for 7 days culture [53]. Andrea et al. combined collagen type I with HA and studied the effect of different bioink formulations of collagen and HA on the capability of bioprinting. The results demonstrated the optimal formulation and the hepatocytes within can be viable for over two weeks [54]. The hybrid of collagen and chitosan was also printed using an extrusion-based bioprinting device and the optimal blending ratio was discovered that can print scaffolds for tissue engineering. The printed constructs can be stable in phosphate buffer saline (PBS) with collagenase for 44 h and had no cytotoxic influence on the 3T3 fibroblasts [55]. Skardal et al. printed the hybrid of collagen and fibrin containing BMSCs and stem cells from amniotic fluid for the healing of skin wounds in mice. It was observed that the cells encapsulated collagen/fibrin fibrous constructs can promote wound closure and re-epithelialization significantly [56].

Alginate or alginic acid is the earliest used biomaterial in extrusion-based bioprinting. It's a polysaccharide with good biocompatibility that is derived from seaweed. Sodium Alginate is the most common water-soluble Alginate which can react with ionic solutions of calcium (Ca^2+^) or magnesium (Mg^2+^) to form hydrogels. The ionic reaction of Sodium Alginate is rapid (within several seconds) and the formed hydrogels have good mechanical strength and biocompatibility [57,58], thus, it is widely used in the field of biomedicine. Researchers studied the influence of different material properties on the printability of the alginate solutions and the human adipose-derived stem cells within showed good viability [57]. Although alginate hydrogels have no toxicity to cells, the disadvantages of slow biodegradability and lacing cell adhesive motifs can limit their further application. Thus, researchers supplemented various natural materials into the alginate to overcome these issues [59]. Gelatin has good biocompatibility, biodegradability, and enriched RGD motif since it is derived from partial hydrolysis of collagen with a triple-helical structure. Gelatin is thermally sensitive and it will change from the status of solid to solution with the increase of temperature. Thus, gelatin is commonly blended with alginate to bioprint a construct with good mechanical strength and biocompatibility simultaneously [60]. Besides, through the modification of gelatin with methacrylate anhydride, gelatin methacrylate (GelMA) can be obtained which is a very attractive polymer for extrusion-based bioprinting since it is thermosensitive, photo-crosslinkable, and mechanically stable. Ouyang et al. printed GelMA microfibers with core-shell, heterogeneous and hollow structures based on in situ crosslinking process [38], as shown in Fig. 3B. GelMA has also been blended with alginate for improved printability (166). Liu et al. used a core/sheath bioprinting nozzle to fabricate GelMA/alginate microfibers, and the alginate as sheath materials can support the GelMA pre-gel solution in the core and minimize the concentration of GelMA (down to 1.5%) to guarantee cells have high viability and can spread [61]. Poly (ethylene oxide) PEO was used to be mixed with GelMA to fabricate GelMA microfibers with porous structure via extrusion bioprinting after the removal of the PEO phase. The porous microfibrous hydrogel exhibited improved cell viability, spreading, and proliferation compared to the nonporous constructs [51] (Fig. 3C). The hybrid bioinks consisting of alginate and cellulose nanocrystal were printed using the extrusion-based bioprinting device to construct a liver-mimetic honeycomb 3D structure. The results verified that the bioprinting process and the alginate/cellulose nanocrystal bioinks showed minimal damage to the encapsulated fibroblast and hepatoma cells [62]. The blending of alginate and methylcellulose with pancreatic islets was demonstrated that can be printed into a macroporous 3D hydrogel construct using 3D extrusion bioprinting. After printing, the islets can retain their viability, morphology, and functionality, and can continuously produce insulin and glucagon [63].

### 2.2 Microfluidic spinning

Microfluidics technique handles or manipulates micro-scaled fluid (with volume ranging from nano-litter to micro-litter) within micro-scaled channels, which is an emerging interdisciplinary involving chemistry, physics of fluids, micro-electronics, biology, and biomedicine [64]. Microfluidic spinning takes advantage of fabricating continuous micro- and nano-scale fibers with diverse geometry, including solid, tubular, flat, grooved, helical, and so forth since it can precisely and systematically regulate the individual fluids and their interfaces [65,66]. In a typical microfluidic spinning device, there's a core fluid and a sheath fluid that flows in contact with each other through two concentric channels. Since the flows will change from turbulence to laminar when the dimension of the fluids decreases to the microscale, the core and sheath fluid won't mix at the interface except for the diffusion. Thus, by designing microchannels with diverse shapes and solidifying the core flow using photopolymerization, chemical or ionic crosslinking, and solvent exchange process, fibers with various shapes can be obtained in the outlet channel.

**Fig. 4:**
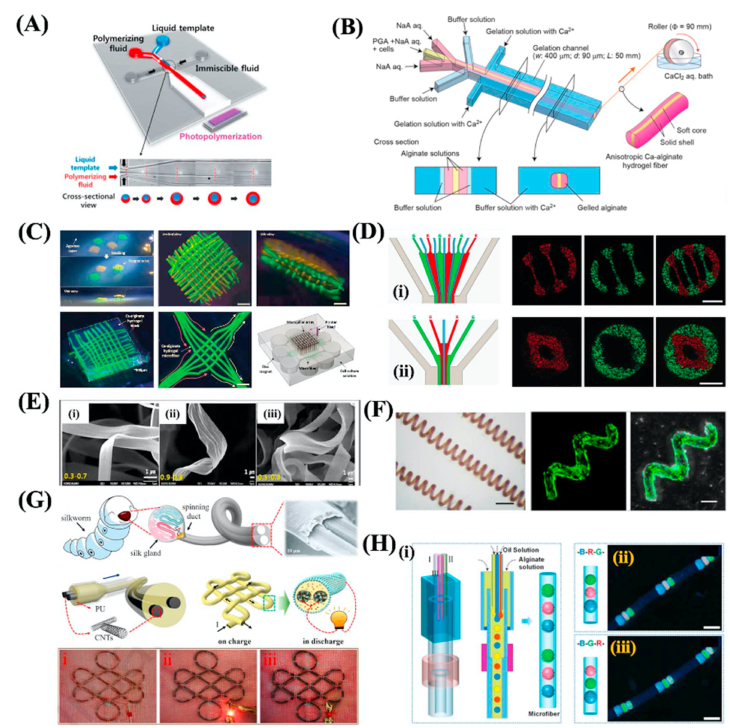
(A) Scheme of the PDMS microfluidic device for the fabrication of hollow fibers. Reprinted with permission from Ref. [67]. Copyright The Royal Society of Chemistry 2011. (B) Schematic illustration showing the microfluidic system for synthesizing anisotropic Ca–alginate hydrogel fibers. Reproduced with permission from Ref. [68]. Copyright The Royal Society of Chemistry 2012. (C) Various microfibrous structures with organized patterns produced via microfluidic spinning. Reproduced with permission from Ref. [69]. Copyright The Royal Society of Chemistry 2020. (D) The schematic and fluorescent images of microfibers with (i) multiple hollows and left-right compartments; (ii) single hollow and multiple shells. Reproduced with permission from Ref. [70]. Copyright 2016 Wiley-VCH Verlag GmbH & Co. KGaA, Weinheim. (E) The SEM images of alginate fibers with (i) thin flat; (ii) seaweed-like; (iii) semi-cylindrical shapes. Reproduced with permission from Ref. [71]. Copyright 2013 Wiley-VCH Verlag GmbH & Co. KGaA, Weinheim. (F) The helical fibers fabricated by microfluidic spinning. Reproduced with permission from Ref. [72]. Copyright 2020 American Chemical Society. (G) The schematic and SEM images of the multi-component CNTs microfibers used as a supercapacitor. Reproduced with permission from Ref. [73]. Copyright 2020 Elsevier B.V. (H) (i) The scheme depicting the fabrication of color-encoded alginate microfibers by microfluidic spinning; (ii) the optical images of the microfibers. Reproduced with permission from Ref. [74]. Copyright The Royal Society of Chemistry 2019. (For interpretation of the references to color in this figure legend, the reader is referred to the Web version of this article.)

Generally, photopolymerization is a process that solidifies the core fluid of the mixture of UV-polymerizable materials and photoinitiator using ultraviolet (UV) light, and polyethylene glycol diacrylate [75], 4-hydroxybutyl acrylate, GelMA [65], polyethylene glycol diacrylate (PEGDA) [76], N-isopropyl acrylamide [77] and glycidyl methacrylate modified dextranare [78] are widely used as the core materials due to their fast polymerization time [67] (Fig. 4A). And the sheath flow in this process is utilized as a lubricant that can ensure the extrusion of continuous fibers without clogging, thus, the viscosity of the sheath flow should be similar to the core flow. The photopolymerization method has the advantages of being simple, controllable, and stable. However, there are limited materials applicable to this method, besides, the UV-polymerizable materials are usually not biodegradable and the UV light may have a negative influence on the sensitive bioactive species that can limit their application in tissue engineering.

For the chemical or ionic crosslinking method, the most widely used core fluid materials are alginate [79] and chitosan [80]. Alginate has good mechanical strength, biocompatibility, and biodegradability, and can rapidly form hydrogels when presented with divalent cations such as Ca^2+^, which make it a very suitable prepolymer candidate for this method [[81], [82], [83], [84]] (Fig. 4B). When fabricating alginate fibers using microfluidic spinning, the sodium alginate solution and CaCl_2_ solution flow into a core channel and sheath channel respectively. In the interface of the two fluids, Ca^2+^ ions rapidly diffuse into the alginate and the sodium alginate will be solidified into fibers and further are extruded out in the outlet channel. Although alginate has many merits, the lacking of cell adhesion motif and the chemical instability can restrict its application. Sodium tripolyphosphate is a commonly used crosslinker for spinning chitosan microfibers [85]. Compared with alginate, chitosan has good cell adhesive and antibacterial properties which are suitable to be applied in tissue engineering. The chemical or ionic crosslinking method has the advantages of materials' biocompatibility and biodegradability. However, the limited mechanical strength can restrict their application.

The solvent exchange is a physical solidification method in which the polymer solution rapidly forms into fibers by precipitation when the diffusion occurs at the interface of solvent and nonsolvent [86]. The choice of precipitant reagent and polymer solution is very essential for the formation of fibers in the solvent exchange method, and the commonly used materials as the polymer solution include poly (lactic acid-glycolic acid) (PLGA) [87], polymethyl methacrylate [88], polybenzimidazole (PBI) [89], polyvinyl alcohol (PVA) [90], regenerated silk fibroin [91] and polystyrene (PS) [92]. Besides, the successful solidification of fibers and regulation of morphology also depends on the suitable choice of solvents and precipitants. Dimethyl sulfoxide (DMSO), N, N-dimethylformamide (DMF), acetone, and tetrahydrofuran (THF) are often used as organic solvents, and PBS, PEO, and ethanol are commonly utilized as the precipitant solution. The solvent exchange method takes the advantage of the wide range of materials choice and controllable physiochemical properties which are determined by the selected materials. However, most organic solutions are toxic which can reduce the biocompatibility of the formed fibers and limit their application in the biomedical field. Besides, since the velocity of the solvent exchange often changes, it's difficult to control the generation process. In all methods, the properties of the formed fibers can be influenced by the properties of the selected materials (including concentrations, viscosities, and surface chemistry) and the flow rate of the fluid. Through the regulation of these parameters and the configuration of the microfluidic device, fibers with diverse geometry, including solid, tubular, flat, grooved and helical, can be obtained.

In general, fibers with solid cylindrical shapes are most commonly and easily fabricated by microfluidic spinning through the coaxial microfluidic chips. Two channels form a cross in the coaxial microfluidic chips, and the inner fluid and outer fluid are driven in the same direction in the cross to form the co-flow pattern. The microscale fibers with cylindrical polymeric structures were firstly fabricated by Jeong et al. using the combination of glass capillary with PDMS [93]. The photopolymerizable (4-hydroxybutyl acrylate (4-HBA) solution and PVA solution were chosen as core fluid and sheath fluid respectively. Alginate is often used to fabricate cylindrical microfibers based on the ionic crosslinking with CaCl_2_ solution [94]. However, the poor mechanical properties of the calcium alginate microfibers can limit their application in tissue engineering. Recently, Sr^2+^ was demonstrated that had more crosslinking cites with alginate molecule than that of the Ca^2+^, which further resulted in the fabrication of microfibers with more robust mechanical performance [95]. Besides, the mixture of carbon nanotubes and sodium alginate solution were used as core flow, and the resultant microfibers exhibited ultrahigh mechanical strength [96]. Cylindrical microfibers can also be weaved into a fibrous construct, Sun et al. fabricated GelMA microfibers with magnetic nanoparticles via microfluidics spinning, a magnetic template was used to arrange the microfibers to form a microgrid-like construct [69] (Fig. 4C).

Microfibers with tubular shapes have been widely applied in materials science and tissue engineering since they have mechanical flexibility and bionic tubular structure, which can promote the linear cell to aggregate, the formation of cellular scaffolds to bio-mimic various tissues, including muscle, blood vessels, nerve, and hepatic-cord structures [70,75,97]. Yu et al. used the technique of soft-lithograph to assemble a hierarchical microfluidic chip, consisting of a core-flow channel, a branched sample flow channel, and a Y-shaped sheath flow channel. Based on the designed device, calcium alginate with multiple hollows and compartments was fabricated [70] (Fig. 4D). By using microfluidic spinning, flat microfibers can also be easily fabricated. Microfibers with special shapes, such as flat and grooved, can also be fabricated using the technique of microfluidic spinning, which is very difficult for other fiber fabrication techniques. For instance, Chae and colleagues proposed the construction of crystal-like ordered and ultrathin flat microfibers. Inspired by the spinning mechanism in the silkworm, they assembled a PDMS microfluidic chip with a sheath flow and a core flow, and the sheath fluid and core fluid were isopropyl alcohol containing CaCl_2_ and sodium alginate solution respectively. Because of the low interfacial tension, the core streams can be easily deformed by regulating the core and sheath fluid flow rate, and flat microfibers were obtained at low flow rates [71] (Fig. 4E). Grooved fibers can mimic the extracellular matrix environment of the human tissues or organs with aligned fibrous structure, and thus they can supply topological cues and promote the adhesion, proliferation, and aligned growth of the cells. Ebrahimi et al. used a simple microfluidic device made of PDMS with a grooved structure to successfully fabricate GelMA hydrogel microfibers with grooved patterns. The grooved microfibers can induce the aligned growth of C2C12 myoblasts and promote the formation of myotube [98].

Due to the vessel- and DNA-like geometry, helical fibers exhibit potential and feasibility in the field of biomedical engineering [99]. In microfluidic channels, the fabrication of helical fibers relies on the “liquid rope-coil effect”. Liu et al. used this effect and a 3D printed coaxial microfluidic spinning nozzle to fabricate helical calcium alginate microfibers, and they first studied the influence of the component ratio of alginate on the formation and characterization of the helical fibers except for the flow rates and solution concentrations [100]. Yu et al. used a capillary microfluidic chip to fabricate helical alginate/PEGDA microfibers, which were used as micromotors by encapsulating magnetic nanoparticles within the fibers. The NIH 3T3 cells were inoculated on the helical microfibers and they can adhere to the surface of the fibers and maintain high viability for 5 days of culture [72] (Fig. 4F). In addition, the fabrication of helical microfibers with core-shell, Janus, triplex, and even double-helical structures have also been demonstrated [101,102].

Besides, microfibers with more complicated configurations have been fabricated using microfluidic spinning [[103], [104], [105]]. For example, Huang et al. fabricated microfibers with curvature-adjustable morphology through programming flow and reaction kinetics in microchannel [106]. Inspired by natural silkworms spinning and their hierarchical fiber structure, Guo et al. generated multicomponent microfibers with double carbon nanotubes cores and polyurethane shells using a multi-channel co-flow microfluidic device. It was demonstrated that the microfibers can be used in energy storage and supply powers for LED lights [73] (Fig. 4G). Meng et al. generated alginate microfibers with ordered colloidal crystal microdots. They assembled the glass capillaries-based microfluidic device with the inner fluid of trimethylolpropane ethoxylate triacrylate (ETPTA) and the outer fluid of alginate solution. The ETPTA would emulsify into microdroplets within the alginate solution, and after crosslinking with Ca^2+^, the alginate with microdroplets would solidify into microfibers. Then, the ETPTA microdroplets were solidified and formed dot-in-line patterns reminiscent after being exposed to UV light. The microfibers with microdots were demonstrated that can be used in the color encoding system [74] (Fig. 4H). Similarly, microfibers with necklace-like knots and bamboo-like structures were generated respectively [28,107].

### 2.3 Wet spinning

The technique of wet spinning has been developed to fabricate fibers used in the industry since the 1930s and is currently followed with interest by scientists and researchers. In wet spinning, a polymeric solution is squeezed into a coagulation bath to form long fibers based on a non-solvent-induced phase inversion process. As shown in Fig. 2, the polymer is dissolved in a solvent, and the nonsolvent for the polymer in the coagulation bath can lead to the removal of the solvent and further result in the precipitation of the polymer into consecutive fibers. The diameter of the fiber ranges from tens to hundreds of micrometers. The wet spinning equipment for laboratory research is usually comprised of an extruding syringe with the flow rate controlled by a pump and a needle equipped on the syringe that is soaked in a coagulation solution. Wet spinning fibers have been produced by natural materials, like collagen, chitosan, and silk ﬁbroin, and synthetic materials, like chitin, polycaprolactone (PCL), and PLGA, and the combination of both such as collagen/alginate, calcium phosphate cement/alginate, and chitosan/tripolyphosphate. In recent years, several emerging materials such as graphene oxide, Mxene, and carbon nanotubes have been added into the polymer solution and wet spun into composite fibers, which can impart the fibers with advanced functions, including enhanced mechanical strength, electrical conductivity, and sensing.

The choice of the solvent and nonsolvent coagulation solution is very essential for the properties and cross-section morphologies of the wet spinning fibers. Take the synthetic material PCL spinning as an example, the most widely used solvents include acetone, N, N-Dimethylacetamide (DMAc), and chloroform/tetrahydrofuran. The corresponding coagulation solution in these systems mainly includes methanol and ethanol [[108], [109], [110]]. For the natural material silk spinning, 1,1,1,3,3,3-hexafluoro-2-propanol (HFIP), N-methyl morpholine N-oxide (NMMO), and hexafluoroacetone (HFA) are mostly chosen as the solvent, and similarly, methanol and ethanol are also the most widely used materials for the coagulation agent [111]. Besides, the processing parameters during the spinning can also influence the fiber performance, including the extrusion rate of the polymeric solution, the air gap between the nozzle and the coagulation bath. In addition, the processing after spinning is also crucial for the function improvement of the fibers. For example, Chiara Rinoldi et al. fabricated cell-loaden hydrogel fibers by wet spinning that were further collected by a rotating drum to form highly aligned hydrogel yarns. The post drawing collecting process can decrease the diameter of the fibers, and the aligned orientation of the fibers resulted in the aligned growth and the specific function expression of the cells [112].

**Fig. 5:**
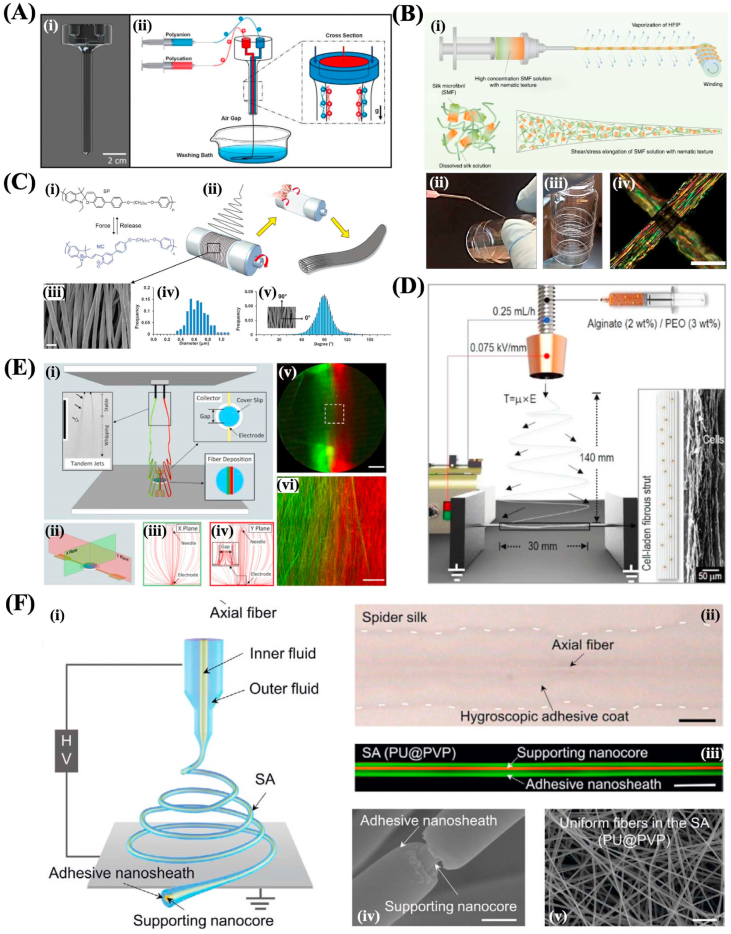
(A) The schematic diagram of the wet spinning device. Reproduced with permission from Ref. [114]. Copyright 2020 The Authors. (B) The scheme (i) and photograph (ii) of the dry spinning process. The optical (iii) and microscopic (iv) images of the fibers. Reproduced with permission from Ref. [118]. Copyright The Authors 2017. (C) (i) The chemical structure of the material; (ii) the schematic diagram of the electrospinning process; The SEM image (iii), diameter (iv), and angel distribution (v) of the fibers. Reproduced with permission from Ref. [119]. Copyright 2018 Wiley-VCH Verlag GmbH & Co. KGaA, Weinheim. (D) The fabrication of cell-laden fibers by electrospinning. Reproduced with permission from Ref. [120]. Copyright 2018 Wiley-VCH Verlag GmbH & Co. KGaA, Weinheim. (E) The schematic diagram of the electrospinning process and the fluorescent images of aligned fibers. Reproduced with permission from Ref. [121]. Copyright 2020 IOP Publishing Ltd. (F) The fabrication of electrospun fibers with the wet adhesive property. Reproduced with permission from Ref. [122]. Copyright 2021 Wiley-VCH Verlag GmbH & Co. KGaA, Weinheim.

The versatility of wet spinning has enabled the fabrication of fibers with diverse morphologies, especially the fibers with core/shell structure, which can enhance the function of fibers by encapsulating various bioactive materials, such as growth factors, antibiotics, and drugs [113]. The wet spinning of fibers with core/shell structures usually involved a coaxial-needle extrusion system to simultaneously extrude two polymeric dopes into the coagulation bath, specifically, when we extruded air or a non-solvent into the inner needle, the hollow wet spinning fibers can be obtained. Research by Qing Cui et al. demonstrated the wet spinning of core/shell chitosan/polystyrene sulfonate (CHI/PSS) fibers with a coaxial nozzle, as shown in Fig. 5A. The extension can stabilize the formation of core/shell fibers and avoid the formation of the beads. The resulting CHI/PSS fiber exhibited excellent mechanical strength and demonstrated its potential as a scaffold for tissue engineering [114]. Meri J. Lundahl et al. investigated the continuous fabrication method for cellulose nanofibrils/guar gum (CNFs/GG) core/shell fibers by wet spinning and post drawing process. They studied the influence of the coagulation ratio in different coagulation agents on the properties of the fibers, and the results demonstrated that higher coagulation speed can endow the fibers with a better orientation ratio and mechanical strength [115]. In addition, hollow wet spinning fibers have also been fabricated by various polymers, including cellulose acetate, polyethersulfone, and biodegradable polymers, such as PCL and PLGA [116,117].

### 2.4 Dry spinning

Dry spinning is another fiber-producing technique, in which the polymer is firstly dissolved in a volatile solvent, like ether or acetone, the polymeric dope solution is then extruded into a spinning chamber followed by the extrusion process [118] (Fig. 5B). The dope solution is forced through the nozzle to form a jet which will subsequently be exposed to a gas environment. With the evaporation of the solvent in the air stream, the concentration of the polymer solution in the filament gradually increases and finally, the filament solidifies into fibers without further drying process. The formed fibers are then drawn by a rotating roll to be collected. In general, the gas used in the dry spinning process is air or inert gas, such as nitrogen. The preparation of the polymeric solution is very essential to the spinning process, the solution usually is filtered before spinning to attain the right consistency. In previous studies, solution concentrations ranging from 15% to 40% have been tried with relevant viscosities ranging from 300 to 5000 P. During the dry spinning process, various parameters can affect the fiber morphologies and properties, including solution properties (e.g., molecular weight, which has an essential influence on the viscosity and solidification rate of the dope solution) and processing variables (e.g., stress, solidification temperature, and mass transfer). Besides, the post-treatment after spinning also has an important effect on the fiber properties [123].

Many polymers have been verified that can be fabricated into fibers via dry spinning, including cellulose acetate, PLLA [124], polyvinyl chloride (PVC), graphene, acrylics, gelatin [125], and silk fibroin. For example, graphene oxide was firstly dry spun by dissolving in the mixed solvent of methanol, ethanol, acetone, and tetrahydrofuran which had high volatility and low surface tension by Tian et al. The obtained graphene oxide fibers exhibited good flexibility and high toughness up to 19.12 MJ m^−3^ [126]. Zhang et al. fabricated hybrid fibers of regenerated silk fibroin and graphene oxide by dry spinning. The results demonstrated that the existence of graphene oxide could greatly influence the viscosity of silk fibroin solution, and the content of β-sheet in the hybrid fibers was lower than that in the pure silk fibroin fibers. Besides, the hybrid fibers exhibited a smaller crystallite size of silk fibroin and enhanced mechanical strength [127]. Apart from these known materials, several newly designed polymers were also be tried via dry spinning. For instance, Masoumi et al. synthesized a novel polymer poly-4-hydroxybutyrate (P4HB) and spun it into a fibrous scaffold with controllable porosity, fiber size, and degradation rate. Besides, the dry spinning fibrous scaffold exhibited good biocompatibility and could induce tissue formation [128]. Overall, due to the difference in the fiber solidification method, dry spinning is different from wet spinning, but the nano- or micro-scale fibrous structure can also impart it with various application in the biomedical field.

### 2.5 Electrospinning

Electrospinning is a versatile process that can continually fabricate nano- or micro-scale fibers via electrostatic force, which was firstly developed by Formalas in 1934. Conventional electrospinning device usually comprises a high voltage power, a needle that is connected to the voltage power, and a fiber collector that was grounded and used to collect the formed fibers. The basic mechanism of electrospinning was shown in Fig. 2. The polymer solution is extruded to the top of the nozzle and firstly forms a suspended droplet. When the high voltage power is applied, the polymer solution will be charged, and the subsequently generated repulsive force will then impel the droplet into the Taylor cone. After the repulsive force fully breaks through the solution surface tension, a charged jet finally transmits from the cone, and fibers are formed on the collector after the repeatedly splitting of the jet and solvent evaporation. Various parameters can influence fiber diameter and morphology, mainly including polymer solution properties (e.g., solution concentration, molecular weight, surface tension), regulatory parameters (e.g., needle gauge, flow rate, voltage, the spacing between the nozzle and collector), and environmental circumstances (temperature and humidity). Among these parameters, the influence of the solution properties is most significant, and the environmental conditions are the least. By controlling these parameters, fibers with diverse morphologies, such as uniform, beaded, or fibers with spindles on a string can be fabricated. Generally, the diameter of fibers is proportionate to the concentration of solution when other factors are the same. An unsuitable concentration may result in the formation of the beads. Besides, the collector architecture and the control over the electrostatic field can also influence the arrangements of the fibers, such as the alignment degree of the fibers. The electrospinning fibers have the merits of high surface-area-to-volume ratio and porous structure, regulatory mechanical properties, great materials handling, easy manipulation of fiber properties, and scalable production, which make them be widely applied in the field of filtration, sensors, catalysis, energy storage, and tissue engineering and regenerative medicine.

After decades of development, a large variety of materials have been explored and fabricated into fibers successfully via electrospinning, including natural polymers (e.g., HA, collagen, silk fibroin (SF), chitosan, etc.) and synthetic polymers (e.g., PLGA, poly-l-lactic acid (PLLA), PCL, PEO, etc.) [[134], [135], [136], [137], [138], [139], [140], [141]]. However, not all of them can be applied in tissue engineering. The materials that can be used for tissue engineering must have excellent biocompatibility. Table 3 shows the polymers widely used as electrospun materials and applied in tissue engineering. From Table 3, single polymers or blended polymers have been used to electrospun fibers for diverse applications, and one material usually can be dissolved in several single solvents or mixed solvents of different components. The components of materials, the morphology of fibers, arrangement of fibers, scaffold modification (such as plasma treatment), biochemical molecules can influence the performance of the electrospinning nanofibrous scaffold.

**Table 3:** The polymers widely used as electrospun materials and their applications in tissue engineering.

Materials	Solvent	Diameter	Morphology	Applications	Ref
Collagen	HFIP/DMF	–	Aligned	Corneal tissue engineering	[142]
Collagen/PLA	TFE/HFIP	300–500 nm	Uniform and bundles	Mimicking of tendon	[143]
Collagen/PCL	HFIP	∼480 nm	Uniform	Wound healing	[144]
Gelatin	Glacial acetic acid	∼450 nm	Uniform	Breast cancer metastatic model	[145]
Gelatin/PANI	DMF/water	–	Aligned	Myotube Formation	[146]
Collagen/HA/PCL	HFIP/Formic acid	–	Hollow	Smooth muscle regeneration	[147]
Silk/PEO	LiBr	200–600 nm	Uniform	Controlled delivery	[148]
PEO/alginate	Tri-distilled water	200–400 nm	Aligned	Skeletal muscle regeneration	[120]
PLGA	HFIP	–	Uniform	Blood−Brain Barrier	[149]
PCL	TFE	∼1.33 μ m	Aligned	Tissue regeneration	[150]
PCL	Methanol and chloroform	–	Hollow	Vascular scaffold	[151]
PLCL/PLLA	HFIP	1.28–1.4 μ m	Aligned and core-shell	Vascular scaffold	[152]
PCL/halloysite nanotube	TFE	0.7–1.2 μ m	Spindle-knot structure	Filtration	[153]
PCL/ALA	HFIP	183–344 nm	Uniform	Wound healing	[154]
PU/MXene	DMF/THF	∼170 nm	Fibrous membrane	Stretchable electronics	[155]
PU/PVP	DMF/EtOH	∼900 nm	Core-shell	Wet tissue adhesive	[122]

Natural materials, including collagen, gelatin, and silk, which take advantage of excellent biocompatibility and biocompatibility, have been extensively used in electrospinning. Zhang et al. electrospun type I collagen nanofibers to construct a small vascular graft. The nanofibrous graft exhibited better mechanical strength than the natural artery and similar radial dynamic compliance to the natural artery. Meanwhile, the collagen graft could promote cell adhesion and proliferation when compared with the synthetic polylactic acid (PLA) graft [156]. G Janani et al. used electrospinning to fabricate gelatin nanofibrous matrix and coated it with collagen for improving surface sophistication and mechanical strength. The scaffold demonstrated better cell adhesion and growth than the uncoated control group and verified the potential to serve as a breast cancer metastatic model *in vitro* [145]. Song et al. based on a colloidal electrospinning technique to construct gelatin nanosphere incorporated silk fibroin nanofibers. The encapsulation of gelatin nanospheres could lead to a more sustained release of biomolecules and could promote the biological function of the silk fibroin nanofibers [148]. Though natural materials exhibit excellent biological properties, due to lacking *in vivo* natural crosslinking agents, the mechanical strength usually cannot attain the requirements and thus restrict their clinical applications. For synthetic materials, although the biological properties are less than the natural materials, they have excellent and regulatory physiochemical properties, which make them also be widely used in electrospinning, especially for FDA-approved materials, like PCL and PLGA. For instance, Qi and colleagues obtained PLGA nanofibrous mesh via electrospinning to establish a novel blood-brain barrier system. The nanofibrous structure could promote cells to proliferate and express the tight junction protein to attain significant barrier integrity and the barrier functions were demonstrated [149]. Rafique et al. electrospun dimethyloxalylglycine loaded PCL nanofibrous scaffold to construct small vascular grafts. The PCL nanofibrous scaffold realized the sustainable release of dimethyloxalylglycine and could promote human umbilical vein endothelial cells to proliferate, migrate and produce pro-angiogenic factors [151].

Besides the single polymer electrospinning, the blended polymers can also be electrospun to modulate the biological and physiochemical capabilities of the electrospinning fibrous scaffold. In general, a blended electrospinning scaffold can be obtained by mixing natural material with synthetic material to integrate the remarkable biocompatibility of natural polymer and the superior mechanical stability of the synthetic polymer. As an example, collagen and HA, PCL was co-electrospun into tubular nanofibrous scaffolds to construct biomimetic vascular grafts. The slow degradation can realize the long-term transplantation of the graft *in vivo*, the addition of HA could induce the migration and differentiation of smooth muscle cells, and promote the regeneration of vascular smooth muscle [144]. Sensini et al. used electrospinning to fabricate collagen/PLLA composite nanofibrous bundles applied in tendon tissue engineering. The bundles exhibited promoted Young's modulus and good cell adhesion and viability [143]. Except for the adjustment of polymer compositions, the blends ratios of different components can also regulate the physiochemical properties of the scaffolds, such as mechanical properties and degradation rate. For example, different ratios of poly (glycerol sebacate) (PGS) and PCL (1:1, 2:1, 3:1, and 4:1) nanofibrous scaffolds were fabricated by electrospinning, and the results demonstrated that Young's moduli increased with the decrease of PGS content. The 4:1 composite nanofibrous scaffold was determined to well match with the mechanical strength of the native corneal tissue [157].

The alignment of the fibers can also impart the electrospun scaffolds with unique properties, such as optical, mechanical, and electrical properties, and enhanced guidance on the orderly growth of cells for several specific human tissues or organs with an aligned extra cellular matrix structure, including nerve, cornea, and tendon, etc. Conventional electrospinning equipment can only result in chaotic fibrous scaffolds, but aligned fibers can be obtained by designing collectors with a specific configuration, controlling the electrostatic field, or adding an auxiliary magnetic field [158]. For example, a rotating cylindrical roller is the most widely used method to develop aligned fibers, as shown in Fig. 5C, when the rotating speed of the roller is over a specific value, the deposited fibers will be pulled to form an aligned arrangement [119]. Two parallel electrodes are another choice of a collector to fabricate aligned fibers by the electric field-assisted method. The existence of the electrodes would affect the distribution of the electrostatic field during the electrospinning process and guide the fibers to deposit parallelly between the two parallel electrodes [120] (Fig. 5D). However, due to the sparse interconnection between the thin and anisotropic fibers, the electrospun membrane fabricated by the electric field-assisted method is usually mechanically unstable. A study by Han et al. exhibited the fabrication of a Janus membrane with aligned fibers and random fibers based on a metal–electrolyte solution switchable collector that can solve this issue [159]. We previously reported a novel design of collector that combined the principle of the rotating cylindrical roller and parallel electrodes, which was the double parallel rotating thin plates [160]. Besides, Wang et al. developed a facile strategy to fabricate nanofibrous membranes with well-aligned structures. They used a nonconductive glass sheet as the collector which was located between the needle and a static drum, and aligned fibers could be obtained before they arrived at the conductive drum [161].

Recently, research interests in electrospinning mostly focus on the fabrication of fibers with special structures except for the alignment of the fibers, such as heterogeneous nanofiber patterns or fibers with beaded structures. For instance, Wieringa et al. developed a novel electrospinning device that can electrospun up to three polymers simultaneously. By controlling the distribution of the electrostatic field using electrodes with either a parallel or epsilon arrangement, they fabricated heterogeneous fibrous membrane in parallel or divergent (‘Y-shaped’) orientations [121] (Fig. 5E). Another study by Zheng et al. also fabricated heterogeneous fibrous membrane based on the multi-nozzle electrospinning. On behalf of improving the control over the fiber deposition, they designed a novel oppositely charge-based device with an air flow to transport neutralized nanofibers, and nanofibers deposited in specific patterns were fabricated [162]. Inspired by the dust capture of spider silk, Huang et al. electrospun PCL nanofibers with a structure of spindle-knot. The formation of spindle-knot was attributed to the halloysite nanotube dispersed in the PCL electrospinning solution. The special structure imparted the scaffolds with synthetic performances of superior surface energy and hydrophilicity [153]. Coaxial electrospinning has been developed in recent years to fabricate fibers with advanced properties and various structures, such as hollow, core/shell, and multi-channeled structures. For example, a hollow carbon microtube was fabricated via coaxial electrospinning with enhanced microwave absorption [163]. Inspired by the wet adhesion of the spider cobwebs, Liu et al. demonstrated the fabrication of polyurethane/poly(vinylpyrrolidone) (PU/PVP, core/shell) nanofibrous scaffolds via coaxial electrospinning. Under the moisture environment, the shell PVP would be dissolved and then could supply interfacial adhesion, and meanwhile the undissolved core PU could supply the intrinsic strength. Thus, the core/shell fibrous scaffolds could maintain reliable adhesion on wet substrates [122] (Fig. 5F). Overall, electrospinning technique is a versatile fiber fabrication method that can generate a large variety of polymer fibers in nano- or micro-scale diameters, and the obtained fibers can be expediently designed into matrices, which facilitate their application in biomedical field.

### 2.6 Near-field electrospinning

The emerging technology near-field electrospinning (NFES) derives from electrospinning and has been widely used in various biomedical applications. In a NFES process, it uses the straight stable jet that appeared in electrospinning by narrowing down the distance between the needle and the collector to avoid the appearance of the spirally unstable process. The equipment of NFES is similar to electrospinning, also includes a high voltage, collecting device, and solution providing system (Fig. 2). But the spacing between the needle and the collecting device is different. The distance is larger than 50 mm for electrospinning, but for NFES, it is smaller than 10 mm. Besides, the collector of NFES is attached to a XY motion platform, but the electrospinning doesn't need. The second difference is the fiber alignment and pattern, NFES can control the printed fibers precisely, and thus the scaffold has a high fiber alignment and we can fabricate fibrous scaffold with any desired pattern. For electrospinning, because it has the spiral instability process, the alignment of the fibers can't be controlled very well; the third one is the fiber diameter, electrospinning can produce fibers with nano-scale very easily, but for NFES, it can produce micro-scale fibers easily as the result of the short spacing between the needle and the collecting device. These features impart NFES with broad applications ranging from tissue engineering to nanogenerators, sensors, and microelectromechanical systems.

**Fig. 6:**
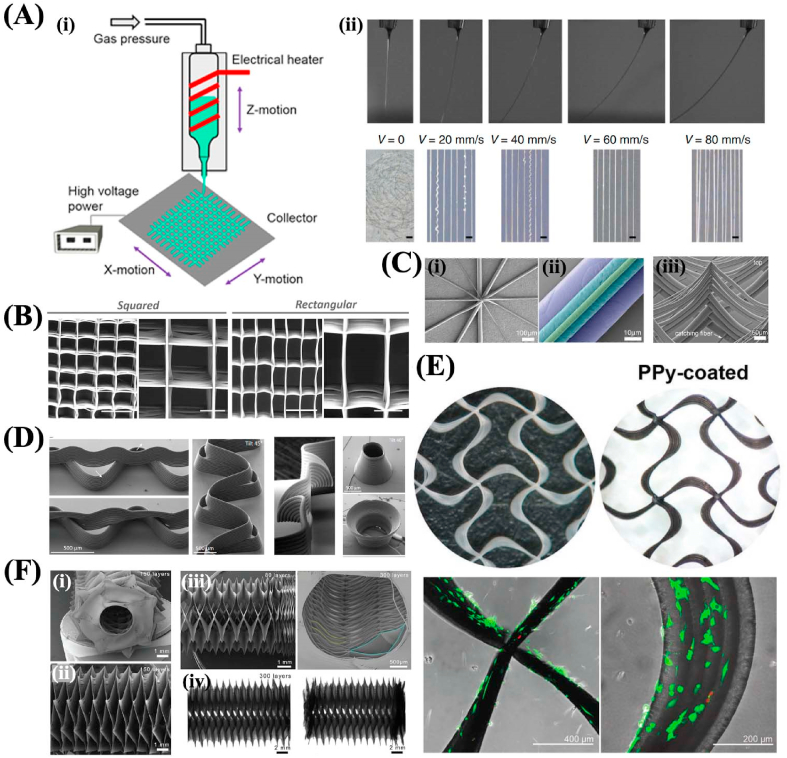
(A) (i) The schematic diagram of the near-field electrospinning process; (ii) the effect of collector velocity on the jet wagging angles. Reproduced with permission from Ref. [5]. Copyright The Authors 2020. (B) The squared and rectangular PCL scaffolds fabricated by NFES. Reproduced with permission from Ref. [129]. Copyright 2017 Wiley-VCH Verlag GmbH & Co. KGaA, Weinheim. (C) (i) The SEM image of NFES fibers with various diameters; (ii) the false-colored SEM images presenting the deposition of fibers with different diameters; (iii) the SEM image of fibrous scaffolds. Reproduced with permission from Ref. [130]. Copyright 2018 Wiley-VCH Verlag GmbH & Co. KGaA, Weinheim. (D) The SEM images of fibrous scaffolds with diverse structures fabricated by NFES. Reproduced with permission from Ref. [131]. Copyright 2020 Wiley-VCH Verlag GmbH & Co. KGaA, Weinheim. (E) The SEM images of NFES scaffolds with wavy shapes and the fluorescent images showing the adhesion of cells. Reproduced with permission from Ref. [132]. Copyright 2020 Wiley-VCH Verlag GmbH & Co. KGaA, Weinheim. (F) The SEM and stereomicroscope images of NFES tubular fibrous scaffolds with different layers. Reproduced with permission from Ref. [133]. Copyright 2021 Wiley-VCH Verlag GmbH & Co. KGaA, Weinheim.

The concept of NFES was first put forward by Sun et al., in 2006. They used a 25 μm tungsten needle to successfully deposit the PEO nanofibers with diameters ranging from 50 to 500 nm on a silicon-based collector in a direct, continuous, and controllable manner [164]. They mainly investigated the fiber fabrication and parameters influence. In general, it has been demonstrated that several parameters during NFES can affect the diameters, morphologies, and alignment degree of the fabricated fibers, including solution concentration, the spacing between the nozzle and collector, applied voltage, and the matched degree of the jet formation speed and collector motion velocity. Apart from these parameters, the nozzle diameter, solution surface tension, and environmental humidity can also affect the deposition and properties of the fibers. It has been verified that the fiber diameters increase with the increased polymer solution concentration, decreased nozzle collector distance, and increased voltage respectively [[165], [166], [167]]. Besides, the matched degree of the jet formation speed and collector motion velocity plays a vital role in the morphology regulation of the deposited fibers. By regulating the matched degree, straight, wavy, and curly fibers can be obtained. Our previous study has demonstrated that the increased collector velocity could result in the increased jet wagging angle, thus the chance of presenting curly fibers decreased. However, the too-large collector velocity could lead to an oversized wagging angle, which could further trigger the fiber depositing trace to deviate from the given path [5] (Fig. 6A).

According to the condition of the polymers, NFES can classify into solution NFES and melt NFES. The polymer is dissolved in a solvent in solution NFES, but for melt NFES, the polymer is heated to the molten phase before spinning without the usage of solvent. Many polymers can be electrospun into fibers via solution NFES, including PVA, PEO, PCL, PS, polyvinylidene fluoride (PVDF), etc. However, for melt NFES, only several polymers have been exploited due to the rigorous requirement for the melting temperature and stability. Although there are many choices for the solution NFES polymers, the existence of the solvent can increase the instability of the electrospinning process which leads to the controllability over the fiber diameter and morphology inferior to the melt NFES. For example, PEO is usually used in solution NFES to fabricate patterned fibers due to its water solubility and good biocompatibility. Various researches based on PEO to study the influence of processing parameters on the fiber properties. While for melt NFES, PCL is the most widely used material due to its low melting temperature, thermal stability, and good biocompatibility. PCL usually was electrospun into the orthogonally aligned square fibrous network, and this structure has been demonstrated that can induce the orderly growth and specific function expression of different types of cells when applied in constructing engineered tissues, such as cartilage, cornea, nerve, and blood vessel [168,169]. However, the fabrication of the fibrous scaffold via melt NFES is limited in the thickness or volume of the scaffold since the charges of the deposited fibers would repel the incoming fibers with the same charge, and further would distort the architectures of the scaffold. To solve this issue, Wunner et al. put forward a novel method, that was maintaining the distance between the nozzle tip and the top surface of the scaffold, and increasing the voltage during the electrospinning, which demonstrated the fabrication of thick (exceeding 7 mm) scaffolds with highly ordered large volume [170]. Apart from the square structure scaffolds, many other special structures were also designed and electrospun via melt NFES. For instance, Castilho et al. electrospun poly (hydroxymethylglycolide-co-ε-caprolactone (pHMGCL) scaffolds with a rectangular structure, which exhibited better induction efficiency of cardiac progenitor cells to grow along the preferential direction than the square scaffold [129] (Fig. 6B). Hrynevich et al. fabricated a fibrous scaffold with diverse discrete diameters (2–50 μm) by controlling the variations of mass flow rate and collector speed. The control over the diameter of fibers during NFES presents a new opportunity for the fabrication of accurate scaffolds for biomedical applications [130] (Fig. 6C). Liashenko et al. supplemented microscale layer shifting during melt NFES via deliberately offsetting the printing trajectory, and fabricated novel nonlinear geometries, including overhangs, wall texturing, and branching [131] (Fig. 6D). Olvera et al. designed and fabricated auxetic PCL nanofibrous scaffolds via melt NFES which could accommodate the strain and stress exerted by the human myocardium during diastole and systole when compared with the square scaffold [132] (Fig. 6E). Mieszczanek et al. studied and analyzed the fiber pulsing occurrence during melt NFES and fabricated collapsible tubular structures [133] (Fig. 6F).

### 2.7 Others

Apart from the above widely used fiber fabrication strategies, some other methods, such as biospinning, direct drawing, and the combination of different technologies are also developed. Unlike other techniques, biospinning is a process that fabricates fibers by insects. Silk fibers can be obtained via biospinning by directly drawing from immobilized insects, and the drawing method can affect the fiber diameters which range from 25 μm to 30 μm for manually drawing and from 65 μm to 70 μm for naturally spun cocoons. Besides, the silk fibrous scaffolds fabricated by biospinning exhibited high porosity and tensile strength [171]. Direct drawing is a process developed under the inspiration of the assembly procedure of natural silk proteins. The occurrence of self-assembly of materials at the interfaces is mainly based on two mechanisms: molecular interactions and interfacial polyelectrolyte complexation. For the latter, two polyelectrolyte droplets with opposite charges are put very closely, and forceps are used to draw in the contact interface to obtain a solid fiber through the polyion complex formation [172]. Continuous fiber has been produced by using a rotating roller from various polyelectrolyte solutions, including chitosan, HA, and sodium alginate. Except for the single fiber fabrication method, many researchers have combined two different methods to construct a more complicated scaffold with enhanced functions. For instance, the microextrusion based 3D bioprinting was integrated with electrospinning to construct a novel 3 D hybrid scaffold, and the bioprinting scaffold could impart the hybrid scaffold with enhanced mechanical properties and structural integrity, while the electrospinning scaffold could mimic the extracellular matrix and support cells to adhere, proliferate and express functions [173]. Ruijter et al. fabricated cell encapsulated aligned micrometer fibrous scaffold by combining microextrusion-based 3D bioprinting with melt NFES in a single-step process. When compared with the conventional two-step fabrication procedure that infuses fibers with hydrogels, the novel one-step fabrication method exhibited a better design of microfiber architectures and cell encapsulation in a controllable spatial organization [174]. A study by Jungst et al. demonstrated the combination of electrospinning and melt NFES to fabricate a hierarchically bilayered tubular scaffold. The outer layer was microfibers with controlled orientation produced by melt NFES to induce the aligned growth of vascular smooth muscle cells, while the inner layer was randomly oriented fibers that could support the formation of a luminal endothelial monolayer [175].

## 3 Applications of functional fibers in the biomedical field

### 3.1 Tissue engineering scaffolds

Tissue engineering was first put forward by Langer and Vacanti and defined as the interdisciplinary field which combines the principle of life science and engineering to construct a biological substitute used for restoring, maintaining, or improving the functions of tissues [176]. As one of the key factors of tissue engineering, scaffolds play an essential role and provide an indispensable supporting environment for cells to adhere, migrate, proliferate and differentiate before the formation of new ECM [177]. The main component of the ECM for major cells *in vivo* is a protein with a nanofibrous structure embedded in the proteoglycan gels, the spatial and temporal fibrous environment can exert a dynamical effect on the cellular behavior by transmitting biochemical signaling cues [178]. Thus, the physiochemical and biological properties of the scaffolds should be matched with natural ECM to better modulate the behavior of the cells, and several general properties should be considered when applied in tissue engineering. Firstly, the scaffolds should have good biocompatibility which can make the scaffolds integrate with the adjacent host tissue after transplantation without the undesired immune rejection response. Secondly, the scaffolds should have good porosity that can allow the in-growth of cells, improve the adhesion of cells, and facilitate the delivery of oxygen and nutrients. Besides, the porous structure can improve angiogenesis when transplanted into vascularized tissue. Thirdly, the scaffolds should have good mechanical strength for facilitating the adhesion, proliferation, and other bioactivities of the cells. Finally, the scaffolds should have regulatory biodegradability that can match the regeneration rate of the new tissues [179]. Inspired by the fibrous network of the natural ECM, nano- or microfibers scaffolds fabricated by the above strategies provide an optimal choice for tissue engineering.

The fibrous materials applied in tissue engineering mainly include natural materials, such as collagen, gelatin, silk fibroin, HA, alginate, chitosan, synthetic materials, such as PLGA, PLA, PCL, and the blend of natural and synthetic materials [180]. Among the fiber fabrication technologies, electrospinning is the most widely used strategy to construct fibrous scaffolds applied in tissue engineering since the nanofibrous production exhibits high porosity and surface area to volume ratio and which can promote cells to adhere, grow and differentiate. Besides, the dimension of the electrospinning fiber resembles the fibrils of natural ECM and has been verified to promote the growth of cells as an effective substrate. Moreover, the physiochemical properties of electrospinning fibers are regulatory, such as the fiber alignment, mechanical strength, and biodegradability, to match the requirements of specific tissues [181]. A large variety of studies have been performed to fabricate electrospinning fibrous scaffolds for constructing engineering tissue, including skin, vessel, cartilage, heart, cornea, and nerve, etc. For some special tissues that the ECM is composed of aligned fibrous networks, such as cornea, nerve, and tendon, aligned electrospinning fibrous scaffolds were fabricated [5]. For example, Apsite et al. fabricated a bilayer scaffold comprising aligned PCL electrospun fibers and random HA fibers to construct a tubular artificial graft to mimic the nerve tissue. The formation of the tubular structure was attributed to the swelling of the random HA fibrous layers in the aqueous solution and the inner aligned layer could provide a guidance to induce the oriented growth of neuron cells [182]. Except for the modulation of fiber structure, certain chemical components are incorporated into the fibrous structure to obtain additional advanced functions. For instance, PCL/gelatin nanofibrous scaffolds incorporated with magnesium oxide (MgO) nanoparticles were prepared via coaxial electrospinning. The addition of MgO demonstrated enhanced activity of alkaline phosphatase and could promote to form the mineralized nodules. Meanwhile, the MgO imparted the scaffolds with high antibacterial properties [183]. Besides, the post-treatment of the electrospun fibers by modifying the fiber surface can also endow the scaffolds with advanced functions. For example, the plasma treatment could improve the hydrophilicity of the fibers to enhance the adhesion of cells; the coating of silver nanoparticles could impart the scaffolds with the antibacterial ability and promote the regeneration of tissues, such as bone [184].

**Fig. 7:**
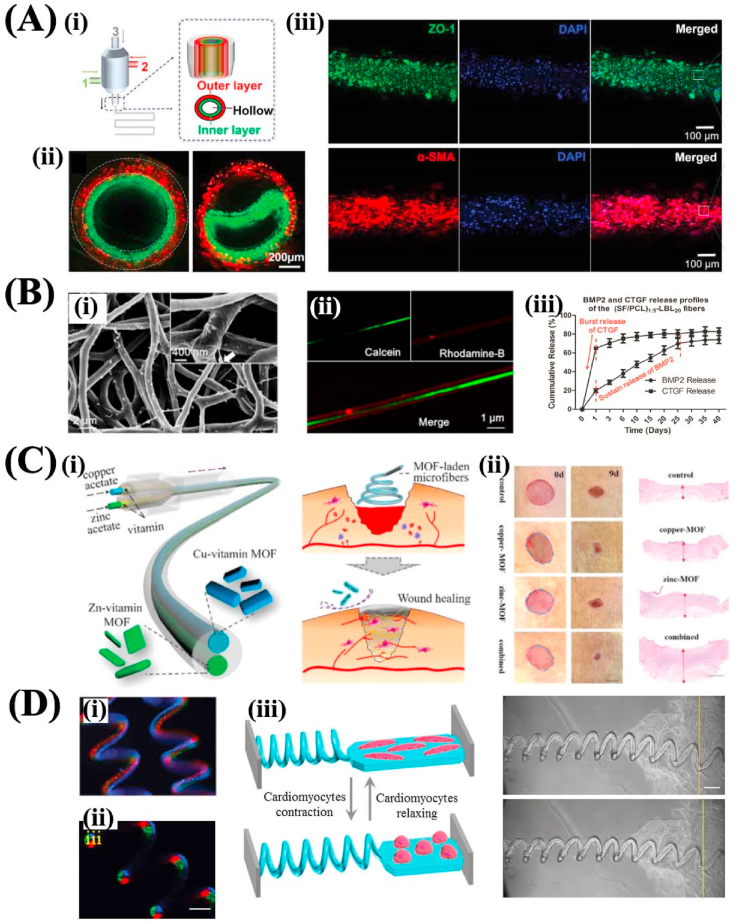
(A) The fabrication of hollow microfibers by microextrusion-based bioprinting applied as urothelial tissue constructs. Reproduced with permission from Ref. [185]. Copyright 2018 Wiley-VCH Verlag GmbH & Co. KGaA, Weinheim. (B) The core-shell SF/PCL/PVA nanofibers fabricated by coaxial electrospinning and the sustainable release of factors. Reproduced with permission from Ref. [196]. Copyright 2019 American Chemical Society. (C) The fabrication of MOF-laden microfiber by microfluidic spinning to improve wound healing. Reproduced with permission from Ref. [213]. Copyright 2018 The Royal Society of Chemistry. (D) The helical microfibers produced by microfluidic spinning applied as magnetically and thermodynamically triggered biosensors. Reproduced with permission from Ref. [102]. Copyright 2017 Wiley-VCH Verlag GmbH & Co. KGaA, Weinheim.

Apart from electrospinning, melt NFES has also been used to construct tissue engineering scaffolds due to its precisely controllable fiber pattern that can simulate the structure of the natural ECM of different tissues [32]. Our previous study has demonstrated the fabrication of orthogonally aligned PCL fibrous scaffold via melt NFES. By regulating the jet formation speed and the collector moving velocity, the deposition of the fibers could be well controlled to achieve the designed patterns. The average diameter of the fibers was about 5 μm. Limbal stromal stem cells could adhere tightly to the scaffold, and the aligned fibers could induce the oriented growth of cells along the direction of fibers and could induce the stem cells to differentiate into keratocytes [5]. Besides, microextrusion-based bioprinting and microfluidic spinning have provided an opportunity to fabricate fibrous structures with the spatiotemporally controllable distribution of cells and biomaterials. For example, a multilayered tubular graft with human smooth muscle cells and umbilical vein endothelial cells was fabricated via the multichannel coaxial extrusion system based on the mixed bioinks of GelMA, alginate, and poly (ethylene glycol) acrylate for mimicking vascular tissues [185] (Fig. 7A).

### 3.2 Drug and gene delivery

Drug delivery is a process that transports various therapeutic drugs to the targeted tissues to alleviate the diseases in a safe way and controllable manner of the drugs amount and treatment time [186]. Generally, the drugs are more easily to be absorbed by the human body when the dimension of the drugs and the materials used to incorporate the drugs are small. Thus, drug delivery with nano- or microfibers has attracted much attention, especially for the electrospun nanofibers with diverse materials/structures, regulatory porosity, and large surface area to volume ratio [187]. The diversity of available biomaterials for the fabrication of electrospun nanofibers can endow researchers with the ability to accommodate physiochemically distinct drugs. The large porosity and surface area to volume ratio can impart the nanofibers with high drug loading/encapsulating efficiency and tunable drug release. The loading of drug molecules by electrospinning can be achieved by coaxial electrospinning, co-electrospinning, multi-jet electrospinning, and fiber surface coating after electrospinning [188].

By coaxial electrospinning, the core/shell nanofibers could be obtained with the drugs encapsulated in the core material. The drug release rate could be well regulated by controlling the component or the degradation rate of the shell material [189]. In co-electrospinning, the drugs were encapsulated in the polymeric solution before spinning. However, there are several challenges to this method, including the influence of the high electric field on the bioactivity of the drugs and the insoluble issue of the drugs in the solvent. The multi-jet electrospinning could produce nanofibers incorporated with several different drugs based on more than two nozzles that could better protect the delivered drug biomolecules [190]. After electrospinning, the nanofibers could also be coated with drug molecules by physical or chemical methods, which can avoid the adverse influence of the electric field or solvent on the drugs. Researchers have demonstrated the coating of drugs through physical electrostatic, hydrophobic, and van der Waals interactions, and chemical modification with amine, carboxyl, hydroxyl, or thiol groups. However, the coating of drugs on the surface of nanofibers can easily lead to the quick release of drugs as there is no barrier between the agents and the surrounding environments [191]. Besides, the microenvironment with non-physiological temperature and PH can weaken the bioactivity of the directly exposed drugs with bioactive components.

The choice of material plays a vital role in achieving sustained drug release from electrospun fibers. Fortunately, as mentioned above, a large number of readily available materials have been explored that can be used for electrospinning. After loading on the fibers, the drug needs to first get through the barrier formed by the fibrous matrices and then diffuse to the surrounding environment. This is the drug diffusing pathway that provides a mechanism to control the release of drugs. The match of a drug with a suitable material, and the alteration of the drug diffusion path are two effective approaches to regulating the release of the drug [192]. Wei et al. fabricated PLGA nanofibrous scaffolds by electrospinning and the anti-inflammatory drug aspirin were incorporated into the fibers by mixing with the PLGA solutions before spinning. The sustainable release of aspirin could enhance the proliferation of BMSCs and induce differentiation to osteogenic cells [193]. Liu et al. produced ferulic acid loading gliadin fibers based on a novel triaxial electrospinning device. The core fluid was the mixture of ferulic acid and gliadin, the middle fluid was the cellulose acetate (CA) solution that can deposit on the gliadin fibers to form a continuous coating of CA. The coating of CA could eliminate the initial burst of release that occurred in the uncoated fibers and resulted in the zero-order releasing profile that can be gradually adjusted by varying the coating thickness [194]. Dodero et al. constructed an alginate/PCL nanofibrous membrane incorporating ZnO nanoparticles via electrospinning. The nanofibers exhibited controllable release of the nanoparticles and could improve cell viability and tissue regeneration [195]. Core/shell structured SF/PCL/PVA nanofibrous scaffolds incorporated with bone morphogenetic protein 2 in the core and connective tissue growth factor modified on the fiber surface were fabricated via coaxial electrospinning. The dual-drug release system demonstrated the sustained release of the protein and growth factor which could improve the regeneration of bone by 43% when compared with the single release system [196] (Fig. 7B).

Microfluidics spinning has also been widely used to fabricate micro-and nano-scale fibers applied in drug delivery since it can precisely and systematically regulate the individual fluids and their interfaces to realize the spatio-temporal control over the encapsulation of diverse drugs. For instance, Huang et al. fabricated ibuprofen and bovine serum albumin (BSA) incorporated bead-on-string microfibers via microfluidic spinning for wound healing. They firstly generated PLA microdroplets in an aqueous phase of sodium alginate by a microfluidic device, followed by the formation of continuous bead-on-string microfibers after the solidification of sodium alginate through the crosslinking with CaCl_2_ in a calcium ion bath. The ibuprofen was encapsulated within the PLA microdroplets and BSA was loaded in the alginate hydrogel phase to endow the microfibers with hemostatic, antibacterial, and cell proliferation functions. The bead-on-string microfibers exhibited controlled drug release capability and could promote the repair of the wound via the encapsulation of drugs [197]. In addition, many researchers have attempted to enhance the efficacy of drug delivery, such as improving the loading content of drugs and the responsively releasing of drugs. For example, Ahn et al. put forward a novel approach to enhance the property of drug entrapment within the microfiber fabricated by microfluidic spinning. Alginate/ampicillin aqueous solution was used as core flow, and low-polarity isopropyl alcohol (IPA) was chosen as sheath flow instead of a water-based flow. The IPA-based sheath facilitated the highly condensed aggregation of alginate polymer chains which could entrap the drugs within the fibrous matrix as large as possible. The results indicated that the microfibers exhibited better ampicillin encapsulation ability, which endowed the microfibers with better antimicrobial property [198].

Except for the delivery of therapeutic agents, such as lipophilic drugs, hydrophilic drugs, antibiotics, liposomes, and proteins, the delivery system can also be used to transmit genes based on nano/microfibers to achieve controlled release by controlling the component and surface area of the fibers or through extrinsic stimuli, like light, PH and magnetic field. While the topological structure and dimension of the electrospinning nanofibers can enhance cell adhesion, growth and induce tissue regeneration on account of their similarity to the natural ECM, the control over the spatial and temporal delivery of the gene can enhance the cellular production of needed proteins to further improve the cell bioactivity. Besides, the control over the topological structures and dimensions of the electrospinning nanofibers can also enhance the efficiency of transfection and affect the therapeutic release kinetics. The genetic materials are usually loaded on the electrospun nanofibers through immobilizing to the fiber surface or encapsulating within the fiber, which can avoid the shortcomings associated with the systemic or local injection to realize the sustainable gene release [199]. So far, researchers have used electrospinning nanofibers containing viral or non-viral vectors as a platform to deliver genetic materials in the form of viral DNA, pDNA, and siRNA, as well as CRISPR/Cas9 systems. Gu and colleagues fabricated recombinant adeno-associated virus (AAV) incorporated polyester urethane urea (PEUU) and polyester ether urethane urea (PEEUU) core-shell nanofibers based on coaxial electrospinning. The sustainable release of AAV was demonstrated and could improve the cell transduction level and enhance cardiac function [200]. Xi et al. constructed HA/PLA core-shell nanofibers loading with liposomes carrying pDNA IL-4 by using coaxial electrospinning. The release of the pDNA can be well controlled by varying the PH of the system and can improve the expression of anti-inflammatory factors, inhibit the expression of proinflammatory factors in bone marrow macrophage [201]. HA/PLLA core-shell nanofibers encapsulating siRNA-ERK2 were fabricated by Liu et al. and the release of siRNA could improve the transfection efficiency *in vitro* and reduce peritendinous adhesion formation *in vivo* [202]. Zhang et al. electrospun PCL nanofibers and coated self-assembling peptide (SAP) on the fibers through the approach of layer-by-layer. Then RGD modified positively charged SAP and pDNA-CRISPR/dCas9 were absorbed by electrostatic interactions. The sustained release could activate the expression and secretion of GDNF, and further promote neurite outgrowth of rat neurons [20]. Apart from electrospinning and microfluidic spinning, the fibers fabricated by microextrusion-based bioprinting and NFES were also reported and used as drug carriers to realize sustainable drug delivery or gene delivery.

### 3.3 Wound healing

Wound healing is an important process for rapid treatment of the injured tissue or organs, which usually consists of four phases, including hemostasis, inflammation, tissue growth, and tissue regeneration [203]. Thus, an ideal material for wound healing should have the ability of hemostasis, antibacterial, and conformation to the injured area, and can absorb the excess wound fluid and transmit water vapor at a rational rate. Besides, the dressing material should be removed and painless for the patient [204]. Hydrogels are the traditional materials used for wound healing, and silver nanoparticles have also demonstrated effective antimicrobial ability to improve the healing process [205]. Current efforts have focused on the use of nanofibrous membranes as wound healing materials because of the large surface area to volume ratio and porosity, regulatory properties, and versatile functions imparted by the incorporation of various biomolecules, which can well meet the most requirements for would healing [206]. Usually, the pore size of the nanofibrous membrane used for wound healing ranges from 500 nm to 1 mm which is small enough to avoid the invasion and penetration of bacteria to protect the injured area via the capturing mechanism of aerosol particles. And the surface area to volume ratio of the nanofibrous membrane used for wound healing ranges from 5 to 100 m^2^/g which is extremely efficient for the absorption of the fluid [207].

The natural materials collagen, chitosan, alginate, HA, synthetic materials PVA, PCL, PLA, PLGA, and the blend of natural and synthetic materials have been fabricated into nanofibrous scaffolds via electrospinning and demonstrated an effective would healing ability [208]. Collagen is widely used to fabricate nanofibrous scaffolds due to its close structural similarity to the most natural ECM and has been demonstrated to significantly improve the wound healing process and inhibit the contraction of the injured area [209]. Similarly, other natural materials with nanofibrous structures have also been reported to be used as wound healing matrices. However, the poor mechanical strength and restricted clinical applications of these natural materials are still a challenge. Therefore, synthetic materials with good biocompatibility and regulatory physiochemical properties were developed. For instance, PLA was electrospun into nanofibrous scaffolds incorporated with cytokine IL-10 loaded HA nanoparticles. The incorporation of nanoparticles and nanofibers could well realize the controlled release of the cytokine. It was demonstrated that the release of the cytokine was regulatory with different healing phases and thus could better improve the tissue regeneration of the skin [210]. Khan et al. constructed a multifunctional poly (l-lactide-co-caprolactone) (PLCL) nanofibrous membrane by coaxial electrospinning incorporated with ZnO nanoparticles and oregano essential oil, which could sustainably release two bioactive agents simultaneously. The incorporation of the agents imparted the membrane with strong anti-bacterial and anti-oxidant activities. Besides, the controlled release of the agents could improve the epithelialization and angiogenesis, terminated the inflammatory cycle and finally realized the healing of diabetic wound [211]. Guo et al. demonstrated the fabrication of a PCL electrospinning nanofibrous membrane loaded with α-lactalbumin (ALA). When compared with the pristine PCL nanofibrous membrane, the composite scaffold can improve wound healing, promote the secretion of collagens and lower the expression of the scar formation relative to smooth muscle actin [154].

Apart from electrospinning, researchers also reported the construction of fibrous scaffolds based on microextrusion-based bioprinting due to its controllable pore size and precise distribution of cells and bioactive components. A recent study demonstrated microextrusion-based bioprinting of fibrous scaffolds with core/shell structure. With the shell material of GelMA, and core material of succinylated chitosan, the fibrous scaffolds exhibited excellent cell viability, formation of the vessel, and accelerated wound closure efficiency [212]. Besides, the fibrous scaffolds fabricated by microfluidic spinning and wet spinning for wound healing were also reported. For instance, Yu and colleagues demonstrated the fabrication of metal-organic framework (MOF) encapsulated microfibers via coaxial microfluidic spinning. The shell material was alginate, and the core material was a copper- or zinc-vitamin framework. The vitamin MOF incorporated alginate microfiber was generated by crosslinking with calcium chloride solution on account of the laminar flow of microfluidic. Through the controllable release of copper and zinc ions and vitamins, which had the properties of antibiosis and antioxidation, the vitamin MOF encapsulated microfibers can enhance the repair of injured tissues [213] (Fig. 7C). Another study fabricated alginate microfibers via wet spinning based on the precipitation of alginate in a CaCl_2_ bath, and silver ions were encapsulated within the fibers through the ion-exchange reaction and reduced to silver nanoparticles. It was demonstrated that the silver nanoparticles incorporated in microfibers could improve the migration of cells to the injured area, reduce the inflammatory phase and overall accelerate the tissue healing speed [214].

### 3.4 Biosensors

Biosensors are defined as “self-contained integrated devices, which are capable of providing specific quantitative or semi-quantitative analytical information using a biological recognition element” by IUPAC [215]. Recently, fibrous scaffolds have been widely applied as biosensors, especially for the nanofibrous scaffolds fabricated by electrospinning due to their controllable porosity, superior surface area to volume ratio, and flexibility that can promote the selectivity, sensitivity, and sensing of the speed of the biosensors [216]. In general, the biosensors by electrospinning nanofibrous scaffolds were constructed by the electrospinning of functional polymers or post-modification of sensitive materials on the surface of the nanofibers. The functional polymers, including polyacrylonitrile (PAN) and polyacrylic acid (PAA), have been reported to be fabricated into nanofibers via electrospinning with inducing functions, which could directly act as the inducing segments of the biosensors. The biosensors obtained by this method feature simple processing, fast responding time, and good biocompatibility. The post-modification of sensitive materials can be achieved through the physical deposition/coating of the sensitive materials on the nanofibers or chemical crosslinking between the sensitive materials and the nanofibers [217]. Various nanofibrous biosensors have been reported, such as electrochemical biosensors and optical biosensors.

Electrochemical biosensors are usually consisting of a recognition system and a transducer, and electrospinning nanofibrous scaffolds can be used as a support for the recognition units. For example, Zhang and colleagues demonstrated the construction of a PVA nanofibrous membrane with the incorporation of graphene quantum dots (GQDs) to construct electrochemical biosensors. The PVA/GQDs nanofibrous membrane exhibited a high surface area to volume ratio and could determine hydrogen peroxide (H_2_O_2_) and glucose with high sensitivity [218]. Another study also reported the fabrication of PVA nanofibrous membrane by electrospinning for the application of optical biosensors. In this study, the glucose oxidase was encapsulated within the fibers by mixing the PVA solution with glucose oxidase before electrospinning, and glutaraldehyde was then used to crosslink the PVA fibers. The results indicated good response and sensitivity to the glucose solution [219]. Apart from these traditional materials, metal or ceramic nanoparticles were incorporated into the electrospinning nanofibers to enlarge the surface area which can improve the sensitivity of the biosensor systems. Moreover, the addition of the inorganic nanoparticles also could expedite the transfer of the electron for the application of biosensor electrodes. For instance, BCL@MOF nanofibers were fabricated by electrospinning that was adhered to a glassy carbon electrode through the binding by chitosan to construct MOF fiber-based biosensor. It was demonstrated that the biosensor exhibited high sensitivity for methyl detection from 0.1 to 38 μM and the detection limit could lower down to 0.067 μM [220]. Apart from electrospinning, microextrusion-based bioprinting, melt electrospinning writing, microfluidic spinning and wet spinning have also been used to construct fibrous biosensor systems. For example, Grigoryev et al. used wet spinning to fabricate alginate fibers incorporated with single-walled carbon nanotubes. The composite fibers exhibited electroconductivity and sensitivity to humidity, pH, and ionic strength since the covalently crosslinked alginate had pH-tunable water-absorbing property [221]. Another study by Yu demonstrated the construction of helical alginate microfibers by coaxial microfluidic spinning to construct force-based biosensors. As shown in Fig. 7D, through the contraction and relaxing of cardiomyocytes, the helical fibers would be stretched. Thus, the helical fibers can be used as a biosensor to monitor the beating behavior of cardiomyocytes [102].

## 4 Conclusion and future perspectives

In summary, this review mainly presents two aspects. In the first part, we introduced several emerging nano/microfibers fabrication technologies, including microextrusion-based bioprinting, microfluidics spinning, wet spinning, dry spinning, electrospinning, near-field electrospinning, and others, such as biospinning, direct drawing and the combination of different technologies. We summarized the equipment, fabrication mechanism, essential processing parameters and their influence, the most widely used materials, and fibers structures and functions for each technique. Besides, the intrinsic merits and drawbacks of each strategy were also discussed. In the second part, we discussed the application values of these functional nano/microfibers and fiber-based materials in several biomedical fields, including tissue engineering scaffolds, drug delivery, wound healing, and biosensors. The cutting-edge studies in recent five years about these applications were highlighted.

Although recent studies have demonstrated fascinating achievements, these nano/microfibers fabrication technologies and applications still have several limitations. First, the choice and investigation of materials are usually restricted to the conventional and common several natural or synthetic materials, such as collagen, silk, HA, PLA, PLGA, PCL, etc. These materials exhibit good processing properties and can be easily fabricated into nano/microfibers by various technologies with controllable morphologies and multiple functions, however, the intrinsic drawbacks can affect their applications, such as the poor mechanical stability of natural materials and the inferior biocompatibility of synthetic materials. Thus, novel materials with advanced functions that can be processed by the nano/microfibers fabrication technologies still need to be developed. Second, the structure of the fibrous scaffolds cannot meet the requirement of most natural tissues or organs. As we know, the natural tissues or organs have sophisticated structures with a specific arrangement that are consist of various components, and multiple cells distributed in different locations to exert specific functions. Although these fabrication technologies could generate functional fibrous structures, no strategy could exactly mimic the natural tissues or organs. Thus, the processing equipment of these technologies needs to be improved or to develop novel integrated devices that can realize multiple technologies, such as the combination of electrospinning and microfluidics spinning to fabricate fibrous scaffold which can bio-mimic the architecture of ECM and meanwhile can realize the spatio-temporal regulation over the assignment of cells and bioactive components. Third, most nano/microfibers fabrication technologies can only produce two-dimensional fibrous scaffolds. However, 3 D scaffolds can provide a better biomimetic growth environment, topological cues, and stable mechanical support for cells, which facilitate the construction of complex functions. Several textile fabrication methods have been developed, such as weaving, knitting, and braiding, to process the fibers into nonwoven textiles. Thus, the combination of these nano/microfibers fabrication technologies with the textile fabrication methods is highly promising for constructing 3 D fibrous scaffolds for better biomedical applications.

Currently, the translation of most nano/microfibers fabrication technologies to clinical practice is still a large challenge and the translational efficiency is very slow, while it is expected that these techniques will generate great revenue and address various challenging issues in current biomedical productions in the future. On one hand, for clinical research and industrial-scale production, production rates of hundreds of grams or more of nano/microfibers per day are desirable. However, the daily production rates of nano/microfibers by current techniques are usually in the gram range. Fortunately, the electrospinning device with multiple nozzles has been designed and widely used in the industry, which can significantly improve the production rates of nanofibers. Benefiting from this innovation, a novel electrospinning scaffold called ReDura fabricated by Medprin Regenerative Medical Technologies Co., Ltd has realized industrialization and has been approved by the Food and Drug Administration (FDA) and China FDA, which has successfully been used in the clinic for the repair of meningeal rupture. On the other hand, most nano/microfibers usually were only tested *in vitro*, which may result from the high costs and low rates of *in vivo* testing. The future of nano/microfibers is to design novel platforms that are easy to use, cost-effective, less demanding in terms of instrumentation, and compliant with industry standards. There is a need for advanced platforms that can characterize various properties of nano/microfibers in a single system. Platforms that can accurately simulate biological systems are also highly desirable, enabling highly relevant *in vitro* and *in vivo* studies.

## Declaration of competing interest

The authors declare that they have no known competing financial interests or personal relationships that could hav e appeared to influence the work reported in this paper.
